# Hallmarks of social action in the vocal turn-taking of wild common marmosets (*Callithrix jacchus)*

**DOI:** 10.1038/s41598-026-60403-2

**Published:** 2026-07-02

**Authors:** Filipa Abreu, Nicola Schiel, Antonio Souto, Simone Pika

**Affiliations:** 1 Comparative BioCognition, Institute of Cognitive Science, Osnabrück University, Osnabrück, Germany; 2 Programa de Pós-Graduação em Biodiversidade, Universidade Federal Rural de Pernambuco, Recife, Brasil; 3 Laboratório de Etologia Teórica e Aplicada, Departamento de Biologia, Universidade Federal Rural de Pernambuco, Recife, Brasil; 4 Laboratório de Etologia, Departamento de Zoologia, Universidade Federal de Pernambuco, Recife, Brasil; 5 Ozouga Chimpanzee Project, Loango National Park, Loango, Gabon

**Keywords:** Language evolution, Primates, *Callithrix jacchus*, Vocal exchanges, Call types, Intergroup communication, Ecology, Ecology, Evolution, Zoology

## Abstract

Conversational turn-taking, the orderly exchange of signals between individuals, is considered an important component of human communication and language. Comparative studies of vocal turn-taking have often been biased toward a limited number of model species and have usually examined only single call types, restricted features of human conversational turn-taking, or captive animals, thereby limiting broader understanding of turn-taking complexity and its evolutionary trajectory. Here we show that wild common marmosets engage in structured vocal exchanges that share several basic features with human conversational turn-taking. We analyzed 1,245 natural vocal interactions and found that individuals can produce multi-turn exchanges with matched call types. Marmosets used different vocalizations when interacting with group members compared to neighboring groups. These interactions were characterized by flexible turn organization, recurring call sequences, relatively short gaps between turns (approximately 1,300 ms), and structured participation involving specific individuals. Altogether, our findings highlight the complexity of vocal interaction in wild common marmosets, providing a broader view of coordination across the whole vocal repertoire and suggesting that elements of human conversational turn-taking may be present in this species and may be shared, at least in part, with other primates.

**Supplementary Information:**

The online version contains supplementary material available at 10.1038/s41598-026-60403-2.

## Introduction

Human language plays a pivotal role in facilitating communication, fostering cooperation, and expressing thoughts, intentions or beliefs and has been considered as unique in the animal kingdom^1^. However, how language evolved is still a big central question in science^2^ and several hypotheses have been postulated^3^. Researchers have been using different methods to investigate language evolution^4^, with the comparative approach being a useful window to pinpoint similarities and differences between human language and the communication systems of other species^5^. Currently, the two most predominant hypotheses (“Gesture-First” and “Vocal Origins of Language”) suggest different modal origins for early human communication^6^. However, recently Levinson formulated the “Interaction Engine” hypothesis, which postulates that it was not language per se that made human communication possible, but a special capacity for social interaction^7,8^ that plays a foundational role in shaping the evolution and dynamics of human communication within social groups. According to this hypothesis, language may have emerged based on a set of socio-cognitive capacities, termed the “human interaction engine,” including joint attention and shared intentionality^9^. In addition to these capacities, the Interaction Engine also encompasses interactive communicative characteristics such as face-to-face interaction, frequent use of mutual gaze, and the exchange of rapid turns - conversational turn-taking^10^.

This last mechanism was systematically described in the last century by ethnomethodological conversational analysts, who outlined the system and its specific features^10^. For instance, conversational turns are reciprocal, comprise at least two participants, and are characterized by the avoidance of overlaps, maintenance of specific temporal relationships, adjacency pairs (sequences of utterances where the second speaker’s turn is depending on the first speaker’s), and communicative repair (strategies employed to address misunderstandings in communication)^10,11^. Participants hold or share information with one another, and responses are very fast (approximately 100–500 ms), suggesting rapid response planning^12^. More recently, scholars have stressed the universality of these features across languages and cultures^8^. For instance, Stivers and colleagues^13^ investigated temporal relationships in turn-taking interactions across ten different languages and found an average distribution of responses of around 200 ms. Concerning the origin and development of turn-taking, studies on human infants indicated that turn-taking interactions begin around the age of three months, when infants start to coordinate signals with caretakers, such as smiles and gaze^14^. Furthermore, Levinson^8^ suggested that there is some evidence of turn-taking across all branches of the primate order, possibly indicating that turn-taking may be a relatively ancient mechanism of the layered system of language.

Hence, scientific interest in turn-taking abilities of other animals has recently increased, with studies tackling similarities and differences regarding a number of turn-taking features. For instance, studies on several animal taxa, including amphibians, birds, and mammals, have demonstrated the presence of some features also found in human social action during conversations (conversational turn-taking) such as adherence to specific temporal relationships and presence of participation frameworks^15^. Concerning our closest living relatives, the nonhuman primates (hereafter primates), research has focused on structured and reciprocal exchanges in vocal and gestural communication, including two main forms of interaction: signal-action (e.g., A produces a gesture, B approaches) and, to a lesser extent, on signal-signal interactions (e.g., A produces a vocalization, B responds with a vocalization). The existing studies have investigated vocal exchanges in several monkey and ape species (e.g., *Ateles geoffroyi; Hylobates lar*) and gestural interactions in great apes (e.g., *Pan paniscus; Pan troglodytes*)^16,17^, providing evidence of structured temporal relationships (avoiding overlap and presence of short gaps between signal-response; e.g., *Hylobates lar*^18^; *Indri indri*^19^; *Pan paniscus* and *Pan troglodytes*^20^, and adjacency pair-like sequences (matched signal-response; e.g., *Ateles geoffroy*^21^; *Macaca fuscata*^22^; *Pan paniscus*^23^.

Among primates, one well-studied model system are common marmosets (*Callithrix jacchus*)^24–27^. They have, like humans, a cooperative breeding system, and a relatively complex vocal communication system^5,28–31^. Scholars suggested that cooperative breeding may have substantially influenced their communicative complexity, potentially resulting in complex socio-cognitive and general complex communicative abilities, including turn-taking^5,32^. Existing studies show that captive marmosets engage in dyadic and triadic vocal exchanges, using PHEE (long-distance contact calls) and TRILL (shorter, softer contact calls) calls^24,27,33^, with temporal gaps that range from 0.6 to five secs and overlap avoidance^24,25,27,34^(For a detailed description of the vocal repertoire and associated call definitions, see^29,34^, Table S1 and Figure S1). Moreover, concerning development, vocal turn-taking has been observed to start relatively early around the age of eight months and seems to be shaped by caregivers^35^. However, most studies focused on only the PHEE call, captive pairs, and single elements of human conversational turn-taking within cooperative or affiliative dyadic frameworks. To examine whether the turn-taking “rules” proposed by the Interaction Engine hypothesis^7^ extend beyond such contexts and vocalizations, we included vocal exchanges from a broad range of natural interactions. This approach allows a more comprehensive characterization of vocal turn-taking in free-living common marmosets and facilitates comparisons with human social action during conversations^17^.

Therefore, we investigated vocal turn-taking in wild common marmosets to examine (i) the diversity of call types involved, (ii) the social partners engaged in these exchanges, (iii) and the extent to which these interactions share key organizational features with human social action during conversations. For this, we identified which vocal exchanges qualified as turn-taking, analyzed their social context (intragroup vs. intergroup), and evaluated key features of human conversational turn-taking: flexibility, adjacency pair-like sequences, timing of responses, and participation frameworks^17^. Flexibility was assessed via adjustments during vocal interactions, adjacency pairs through predominant signal-response sequences, response timing via interval consistency, and participation frameworks by whether vocalizations were directed to specific individuals or an audience.

## Results

The coding resulted in a total of 3,213 vocalizations, of which 1,245 (39%) received at least one vocal response by another individual. Most of the vocal interactions involved single turn-transitions (677; 75.4%), where one individual produced a vocal turn that was responded to by a vocal turn from a receiver. The remaining interactions were two turn-transitions (159; 17.7%), and equal or more than three turn-transitions (62; 6.9%).

Concerning the investigation of the diversity of call types involved in turn-taking vocal interactions, we found that common marmosets most frequently produced PHEE, both when starting a vocal interaction and when responding (initiator: 657; 53%; receiver: 440; 35%), followed by TWITTER (initiator: 255; 20%; receiver: 446; 36%), and TRILL (initiator: 225; 18%; receiver: 238; 19%). Other vocalizations consisted of eight call types (e.g., CHIRP, SUBMISSIVE CRY), which were used in 9% (108) by the initiator and 10% (121) by the receiver. Moreover, the distribution of response types to the most common initiator call types is summarized in Table 1.

**Table 1:** Distribution of call types produced by the receiver (response call type) to the common call types produced by the initiator (call type produced) as a function of number of responses (*N*) and percentage of responses (%).

Call Type Produced	Response call type	Number of Responses (*N*)	Percentage of responses (%)
PHEE	PHEE	347	53
	TWITTER	198	30
	Other vocalizations (e.g., PHEE_STRING, CHIRP)	23	4
	TRILL	49	7
	TRILL_PHEE	40	6
TWITTER	TWITTER	151	59
	PHEE	42	17
	TRILL	41	16
	Other vocalizations (e.g., TRILL_PHEE)	21	8
TRILL	TRILL	135	60
	TWITTER	55	24
	PHEE or others (e.g., SUBMISSIVE CRY)	35	16

*Please see Table S1 for call descriptions.

Regarding our second aim related to the social partners engaged in these exchanges, the results showed that common marmosets most often engaged in turn-taking interactions with members of their own social groups (670; 54%), whereas vocal interactions with members of other groups occurred rarely (82; 7%). For all other vocal interactions (493; 39%), one of the two interlocutors could not be reliably identified. In these cases, vocal interactions could occur with individuals from neighboring groups, or within the social group when members were not in close proximity (not visible). In some cases, we could identify the group to which the individual belonged, but not the individual. In both within-group and between-group interactions, individuals primarily used PHEES and TWITTERS. However, during interactions with individuals from different groups, common marmosets produced not only PHEES and TWITTERS (141; 86%) but also compound calls such as TRILL_PHEE (10; 6%), TWITTER_PHEE (8; 5%), and TRILL_TWITTER (1; 1%). In contrast, intragroup communication also included CHIRP (16; 1%) and TRILL (397; 30%), along with compound calls (121; 9%), including TRILL_PHEE (88; 7%), SUBMISSIVE CRY (8; 1%) and TWITTER_PHEE (6; 1%). The remaining calls were PHEES (409; 31%) and TWITTERS (396; 29%).

Concerning our third research aim regarding the similarities/differences between common marmosets’ vocal interactions and human social action during conversation, we will present the results in the following paragraphs with regards to each tested element.

### Flexibility of turn-taking organization

**Fig. 1:**
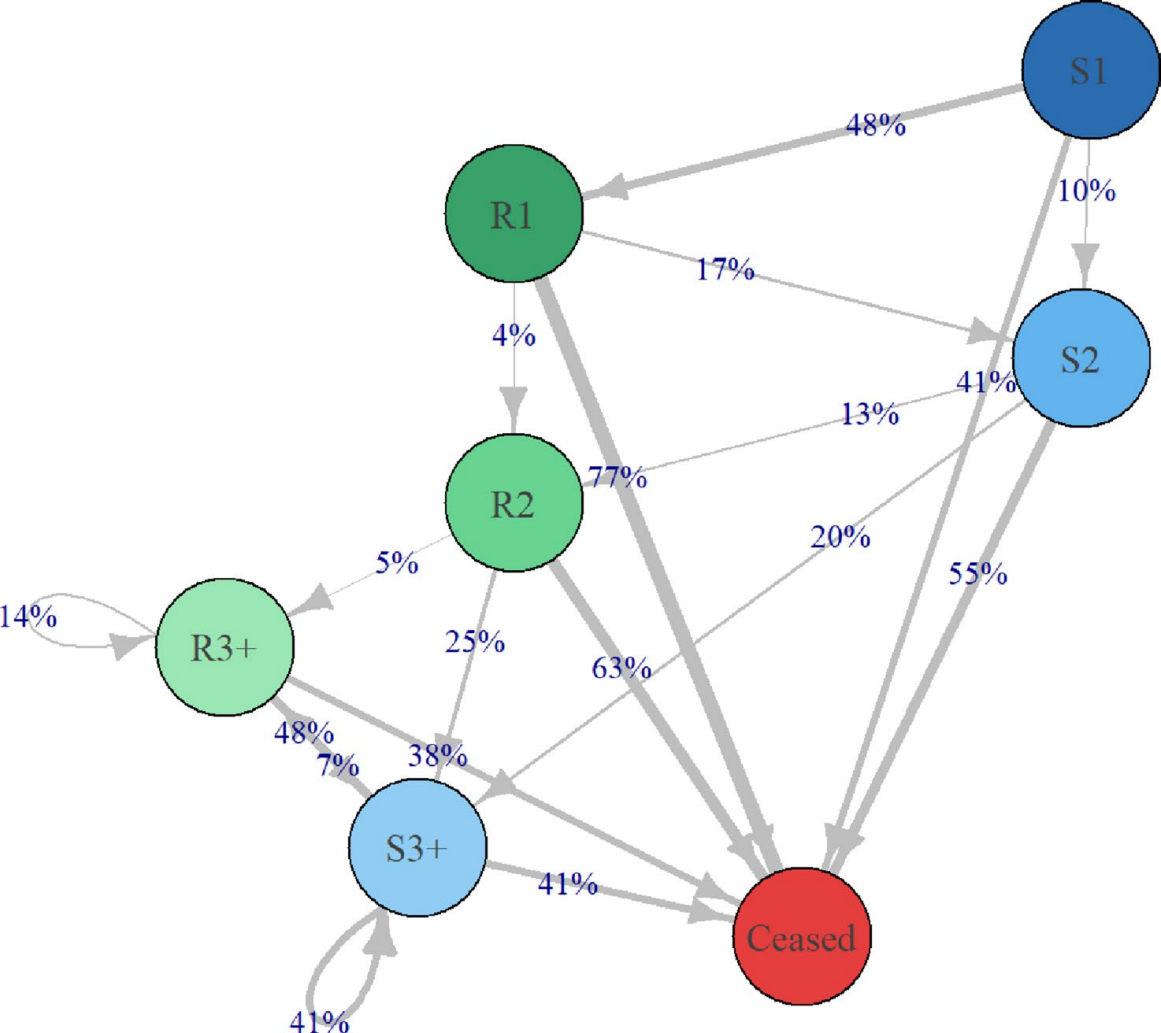
Markov chain probabilities of transitioning from one state to another. States “S1” to “S3+” represent signaling turns by the initiator, and states “R1” to “R3+” represent successive response turns from the receiver within an interaction. “Ceased” refers to an absorbing state representing termination of the interaction. Thicker arrows indicate higher transition probabilities.

To be able to test the presence of flexibility during turn-taking interactions, we investigated both repetition events and turn-transitions. We found that neither the type of repetition (estimate = 0.307, SE = 0.421, z = 0.73, 95% CI [− 0.529, 1.060], *p* = 0.465) nor the number of repetitions (estimate = 0.069, SE = 0.181, z = 0.379, 95% CI [− 0.296, 0.375], *p* = 0.704) had a significant effect on the likelihood of an animal receiving a reply (X^2^ (2) = 0.59, df = 2, *p* = 0.7445). Additionally, within instances of repetition, we found that our study individuals could repeat the same vocal signal (persistence) in 96 cases (69%) and produced a different vocalization (elaboration) in 44 cases (31%). Our results also demonstrated that the longest sequence of call repetitions was eleven when individuals did not hear a response. Regarding the Markov chain analysis, the initial signal (S1) most frequently transitioned to a response (R1; 48%) or to termination (Ceased; 41%), with fewer transitions to a repeated signal (S2; 10%). After R1, the most common transition was termination (77%), while fewer transitions led to additional responses (S2; 17%) or further signaling (R2; 4%). From S2, transitions most frequently led to termination (55%), followed by S3+ (20%) and R2 (13%). From R2, transitions most frequently led to termination (55%), followed by S3+ (20%) and R2 (13%). At later stages (S3 + and R3+), S3 + most frequently transitioned to termination (41%) or remained repeating the signal (41%), with fewer transitions to R3+ (7%). From R3+, transitions most frequently led to another response (48%), followed by termination (38%) and remaining repeating vocal signals (14%) (Fig. 1).

**Fig. 2:**
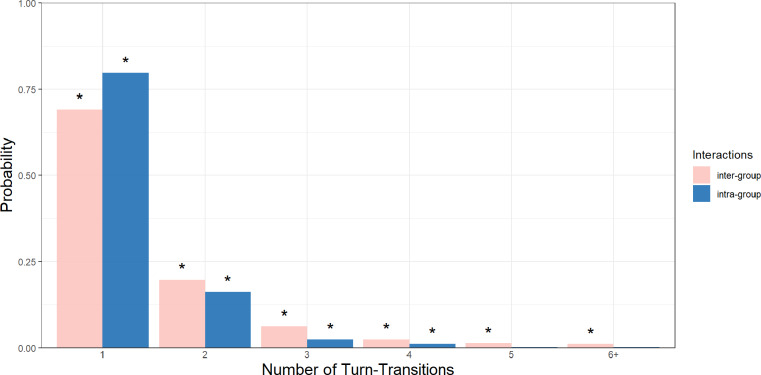
Probability of vocal turn-transitions with one (1) or more than six (6+) turn-transitions for within-group (blue bars) and between-group (pink bars) interactions. Asterisks (*) indicate the vocal turn-transitions that occurred more frequently than expected by chance in inter- and intra-group interactions.

Furthermore, our results showed that one-turn transitions occurred more frequently within groups (intra-group: 422/529, 0.798) than between groups (inter-group: 255/369, 0.691). By contrast, for all other turn-transitions (2–6+), frequencies were higher in inter-group interactions than intra-group interactions (Fig. 2). Moreover, our analysis revealed that most probabilities of engaging in turn-transitions differed from random expectations. For both inter-group and intra-group interactions, most turn-transitions of 1 to 4 occurred more frequently than expected by chance (inter-group: 1-turn, *p* = 0.001; 2-turns, *p* = 0.001; 3-turns, *p* = 0.001; 4-turns, *p* = 0.001; intra-group: 1-turn, *p* = 0.001; 2-turns, *p* = 0.001; 3-turns, *p* = 0.001; 4-turns, *p* = 0.001; Figure S2). However, for intra-group interactions involving five or more turns, observed frequencies did not exceed the expected values (5-turns, *p* = 0.251 and 6-turns, *p* = 0.175, but please see Figure S3, S4 and Table S2).

### Adjacency pair-like sequences

**Fig. 3:**
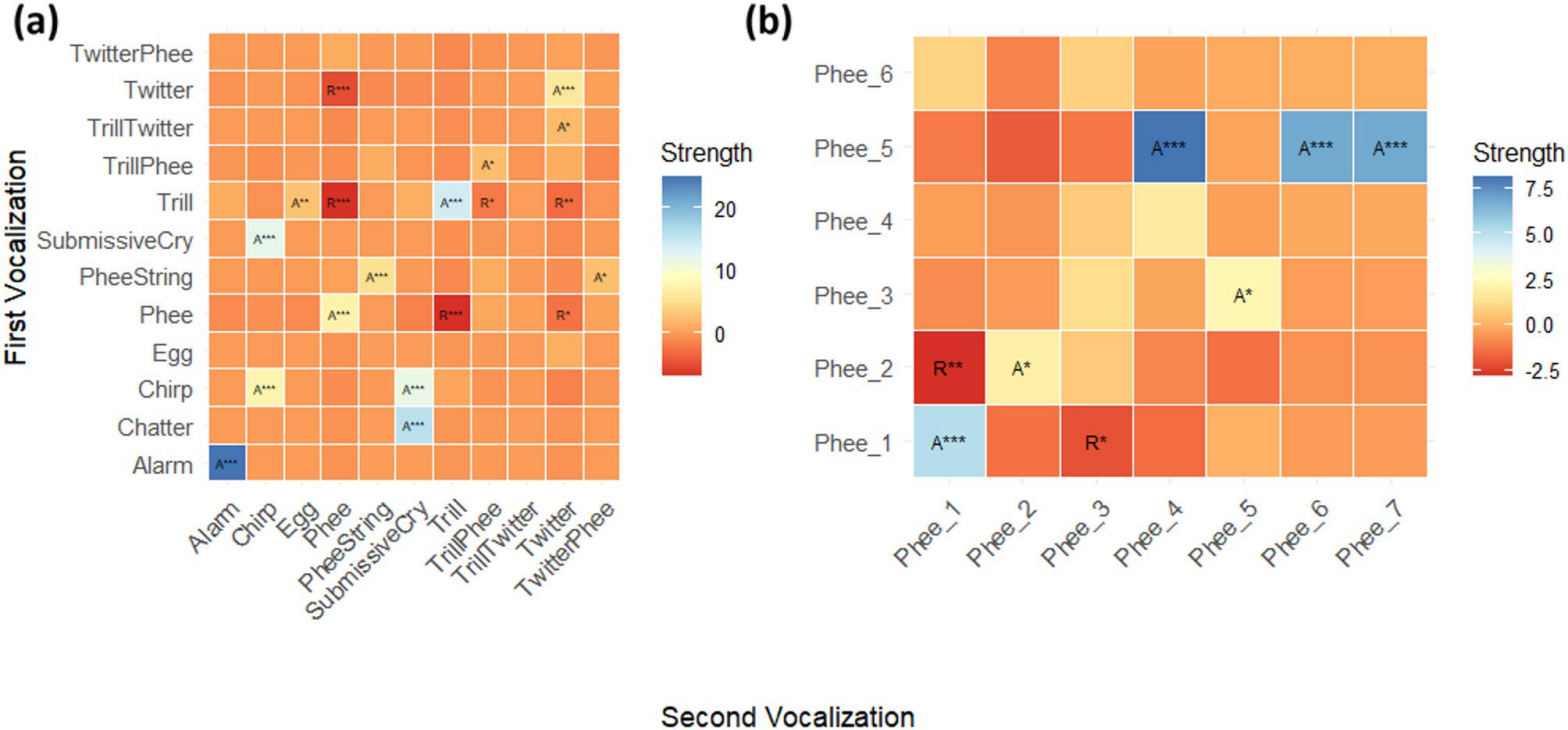
Multiple Distinctive Collocation Analysis based on: (**a**) vocalization pairs, and (**b**) number of syllables within PHEE sequences. Vocalizations from the initiator are displayed in the rows, and responses (receiver’s vocalization) in the columns. The color gradient reflects attraction values, depicting a range from more repulsive pairs (red colors) to more attractive ones (blue colors). Statistical significance of associations is indicated within each cell (A = attraction, R = repulsion), with asterisks denoting significance levels (* *p* < 0.05, ** *p* < 0.01, *** *p* < 0.001).

Our Multiple Distinctive Collocation Analysis (MDCA) results showed that some vocalizations were most often responded to with specific and matching vocalizations compared to others (Fig. 3a). For instance, we found higher levels of attraction between calls used in specific contexts, such as ALARM-ALARM (strength value: 24, *p* < 0.001). We also found high levels of attraction on pairs as TRILL-TRILL (strength value: 14, *p* < 0.001), followed by PHEE-PHEE (strength value: 8, *p* < 0.001), and TWITTER-TWITTER (strength value: 6, *p* < 0.001). Some vocalizations resulted in the production of other vocalizations, such as the CHIRP being positively matched with the SUBMISSIVE CRY (strength value: 11, *p* < 0.001) or also with CHIRP (strength value: 8, *p* < 0.001, Fig. 3a). Conversely, some pairs displayed a higher repulsive value, such as the TRILL-PHEE pair (strength value: −7) and the PHEE-TRILL pair (strength value: −7). We also found that marmosets responded to PHEE vocalizations predominantly by producing the same number of syllables. For instance, a PHEE with one syllable had a high level of attraction with a PHEE with one syllable (strength value: 5.1, *p* < 0.01), and the same result was found for PHEEs with two (strength values - PHEE 2: 1.98, *p* < 0.05). However, PHEEs with five syllables also showed attraction values with other different PHEEs sequences (Fig. 3b).

### Temporal relationships

**Fig. 4:**
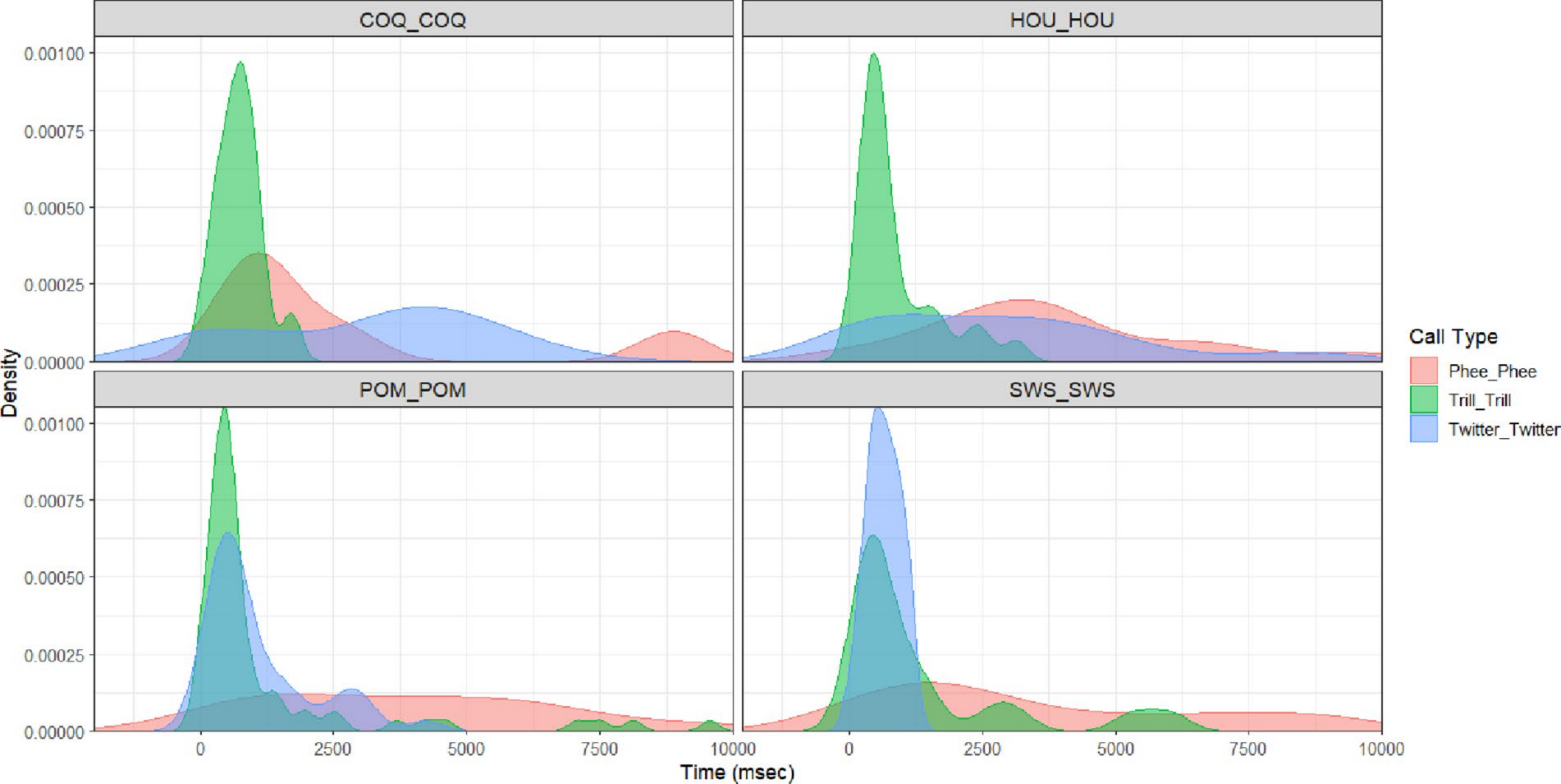
Density distribution of gaps (msec) during vocal interactions within the different groups observed and different vocalization pairs.

We found that the gaps between all turn transitions of common marmosets had a median duration of approximately 1300 ms, ranging from − 2119 ms to 9975 ms (Figure S6), with 3% (40) of all interactions overlapping. Moreover, we found that the full model significantly improved fit compared to the null model (likelihood ratio test: X^2^ (6) = 13.89, *p* = 0.031, Table S4), indicating that call type significantly predicted temporal gaps, with shorter gaps for TRILL-TRILL (estimate = 0.103, SE = 0.015, 95% CI [− 0.134, − 0.073], *p* < 0.001) and TWITTER-TWITTER (estimate = 0.0725, SE = 0.0167, 95% CI [− 0.105, − 0.040], *p* < 0.001) relative to PHEE-PHEE calls, whereas group identity effects were not significant (all *p* > 0.40; Fig. 4). Duration was also not significant (*p* = 0.64).

### Participation framework

**Fig. 5:**
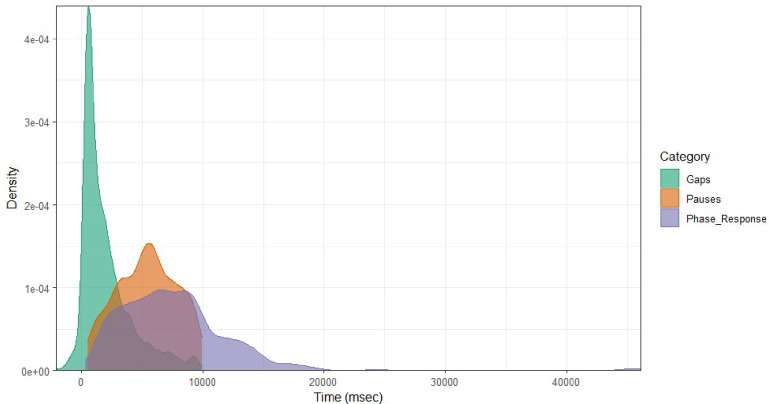
Density distribution of gaps, within-individual pauses and phase responses of common marmosets.

The results showed that the median duration of within-individual pauses was approximately 5,470 ms, ranging from 501 ms to 9,950 ms, whereas phase responses had a median duration of 7,000 ms, ranging from 339 ms to 46,160 ms. All categories significantly influenced interval duration after controlling for call type (likelihood ratio test comparing full and reduced model: X² = 861.14, *p* < 0.0001; Table S5). Specifically, both pauses and phase responses were significantly longer than gaps (Gaps vs. Pauses: estimate = − 2938, SE = 189, t(1413) = − 15.53, *p* < 0.0001; Gaps vs. Phase responses: estimate = − 5229, SE = 162, t(1326) = − 32.27, *p* < 0.0001). Furthermore, phase responses were significantly longer than pauses (Pauses vs. Phase responses: estimate = − 2292, SE = 219, t(1773) = − 10.44, *p* < 0.0001; Fig. 5).

## Discussion

In this study, we aimed to further understand the breadth and complexity of vocal turn-taking skills in free-living common marmosets, a crucial model species to tackle the evolutionary trajectory of turn-taking abilities across the primate lineage, beyond our closest living relatives, *Pan* genus^5^. This study represents the first comprehensive effort to examine turn-taking hallmarks previously defined primarily in human social action during conversations in marmosets living in their natural environment, focusing on both inter- and intragroup vocal interactions. We pursued three primary objectives: (i) characterizing the specific call types utilized during vocal turn-taking; (ii) identifying the social partners (inter vs. intragroup) involved in these exchanges; and (iii) establishing the parallels between marmoset vocal interactions and human conversational systems. Overall, our results revealed that common marmosets living in their natural environment produced different call types to initiate or respond to a vocal turn. Also, they frequently engaged in vocal interactions with members of their own social group but also occasionally during encounters with individuals of neighboring groups. Our analyses further indicated that the observed vocal interactions showed patterns consistent with turn-taking elements such as flexibility of organization, adjacency pair-like sequences, temporal relationships, and participation frameworks.

Concerning our first research aim, we found that although PHEEs and TWITTERs were the most predominantly produced vocalizations, our individuals also emitted other call types to engage and respond to conspecifics. These results contrast to a study that evaluated different call types in captive pairs of common marmosets’ interactions, which showed that TRILLS were the most common vocalizations^33^. However, these differences may be due to the close proximity in which that study was conducted, as the animals were separated by no more than 600 centimeters and always maintained visual contact. Contrarily, our findings align with results from previous research on wild common marmosets in the Atlantic Forest, where it was found that PHEEs and TWITTERs, along with TRILLs, had higher emission rates and versatility across different behavioral contexts^29^. Concerning objective two, we found that our study animals engage in vocal interactions inside and outside the social group, mainly using PHEEs and TWITTERs. However, these two vocalizations were more common during intergroup interactions when compared to intragroup ones, supporting a previous study that reported that both call types are frequently used during intergroup encounters^29^. In addition, when interacting with individuals from their social group, common marmosets produced in addition to those calls, TRILL vocalizations. Thus, our findings seem to be in line with previous studies with wild and captive marmosets, showing that TRILLs are usually emitted within the social group or when both initiator and receiver have visual contact^25,29,33^. In the following paragraphs, we will discuss the findings in relation to objective three and all investigated elements of human conversational turn-taking.

### Flexibility of turn-taking organization

Our findings revealed that common marmosets repeated vocal signals, sometimes producing up to eleven calls, regardless of whether they received a response. While individuals could persist with the same call or used alternatives like PHEEs and TWITTERs, neither repetition type nor number significantly influenced the likelihood of a reply. This pattern contrasts with previous findings showing that elaboration across modalities (gesture-vocal) can increase response rates^36^. However, consistent with earlier work, repetition of different signals but within the same modality (e.g., auditory), here, vocal signals, may instead function to refine the message rather than to increase response probability^37^. Moreover, we also found that responses were most likely to occur early in an interaction, with the probability of a reply decreasing as exchanges progressed^24^. Across vocal interactions, individuals frequently either received a response or ceased vocalizing following their initial signal. Notably, termination became the most common structural outcome after the first response exchange. This pattern suggests that conversational interactions in this species are generally brief, a finding that aligns with our Markov chain transition probabilities and is consistent with rapid turn-taking and early contingencies in marmoset vocal exchanges^24,35,38^. In our study, signal repetition was present but infrequent, as initial calls were usually followed by an immediate partner response. However, when a response was absent, the occurrence of call repetition mirrors patterns observed in other primates. For example, in chimpanzee (*Pan troglodytes*) gestural communication, repeated or elaborated signaling routinely occurs in the absence of an immediate response^36^. Altogether, these patterns are consistent with the interpretation that call repetition may contribute to attention modulation or signal persistence^39–41^, rather than being tightly linked to eliciting a specific type of response. While the underlying mechanisms may differ across modalities and species, these observations suggest that repetition in signaling seems to be a common feature of primate communication systems.

Furthermore, we observed that common marmosets engaged in vocal interactions involving varying numbers of turn-transitions across the study period. One-turn transitions were the most frequent overall, whereas interactions involving multiple turn-transitions also appeared in intergroup contexts (but please see Figure S3, S4 and Table S2). This pattern indicates that, while brief exchanges predominated, longer vocal sequences also occurred, particularly during interactions between groups. These last findings slightly differ from previous primate studies in which signal-signal exchanges were often reported as being limited to a single turn-transition^42^. However, rather than implying sustained interaction as a typical outcome, our results suggest that the length of vocal exchanges may vary depending on social context. One possible explanation for this variation relates to differences in familiarity and spatial arrangement. Within-group interactions often involve individuals in closer proximity and with established social relationships, which may reduce the need for extended vocal exchanges. In contrast, intergroup interactions frequently occur over greater distances and between less familiar individuals, conditions under which vocal exchanges may persist for longer durations^43–47^. However, these patterns should be considered alongside certain methodological constraints of the present study. Because a portion of interactions could not be definitively assigned to known social groups, some recorded events may have captured ambiguous social settings. Restricting the analysis exclusively to fully identified interactions yielded a smaller intergroup sample size, which limited our statistical power for detecting long turn-transitions (please see Table S2). Furthermore, because visibility between interlocutors and the behavioral context were not systematically tracked, the exact functional drivers of these extended interactions remain to be fully understood. Therefore, while the functional significance of these longer interactions cannot be determined from the present data, their occurrence indicates that marmoset vocal turn-taking is not strictly restricted to brief exchanges, suggesting a degree of flexibility in vocal interaction structure.

### Adjacency pair-like sequences

Our findings revealed that individuals consistently responded by producing the same vocalization and same number of syllables within PHEE vocalizations. There was a strong attraction between PHEE-PHEE, TWITTER-TWITTER, and TRILL-TRILL pairs, while PHEE-TRILL and TRILL-PHEE pairs were rarely observed together. However, we also observed vocalization matching across different types. For example, CHIRP, which conveys food-related information^45^, was paired with a CHIRP and a SUBMISSIVE CRY. Taken together, these patterns indicate the presence of non-random call-response sequences in common marmoset vocal interactions, with certain call types reliably eliciting specific responses. These sequences likely facilitate coordination of social interactions and maintenance of proximity, consistent with prior findings in other studies. For instance, comparable paired sequences have recently been documented in wild chimpanzees, where mother-infant interactions in joint leaving and nursing contexts exhibit non-random adjacency pair-like sequences and flexible turn-taking organization^48^. Similar results have additionally been reported in other studies on signal-signal and signal-actions exchanges in human and non-human primates^11,20,23^. Moreover, observations from both captive and wild marmosets similarly report predominant PHEE–PHEE pairings^35^ and frequent TWITTER–TWITTER responses^29^. Overall, these findings highlight that such capacity for sequence organization is not unique to one primate species.

### Temporal relationships

Our findings suggest that marmosets tend to respond relatively quickly and show a low frequency of overlapping vocalizations, consistent with previous work on captive populations^24,27^. Observed gap durations fall within ranges reported for captive TRILL (629–1000 ms^25^) and PHEE calls (5000–5630 ms^24,41^), although slight differences may have resulted from the inclusion of multiple call types, social contexts, and spontaneous interactions in a natural environment^29^. Methodological differences, such as our use of a 10-second cut-off to define interactions, compared to longer windows in previous studies, and the inability to include behavioral contexts or account for visibility, may also have played a role. However, shorter cut-offs have been shown to not prevent frequent exchanges^29^. The distribution of gaps in our study broadly aligns with observations from free-ranging monkeys^21,49^, wild great apes^50^, and closely match a recent study on gesture-gesture turn-taking in wild chimpanzees (−1600 to 8640 ms across five communities)^42^.

Furthermore, our findings provide additional support for a shared turn-taking system within the species, as we found no differences in gap intervals across group memberships. Similar regularities in response timing have also been described in other taxa, including birds and mammals, where antiphonal calling and chorusing follow stable temporal rules^46,47,51^. Importantly, such consistency in response timing resembles the temporal organization in chimpanzees^42,50^. While these patterns are also similar to human conversational turn-taking^13^, the typical response intervals we observed (~ 1300 ms) are substantially longer than those in human conversation (typically 200–300 ms). Nonetheless, these findings suggest that structured response timing can emerge across species, potentially supported by different underlying mechanisms. Within this shared temporal framework, we nevertheless found significant differences between vocalization types: response intervals following TRILL and TWITTER calls were shorter than those following PHEE calls, indicating that the temporal dynamics of turn-taking in this species seems to be modulated by call type. This pattern aligns with previous observations in captive marmosets, where TRILLs are typically shorter and exchanged more rapidly than PHEEs^25^. These differences likely reflect the functional roles of the calls and the spatial context of their use, with TRILLs and possibly TWITTERs facilitating rapid, close-range social interactions and more PHEEs serving longer-distance communication^29,52,53^. Overall, our findings suggest that marmosets adhere to the rule of overlap avoidance and demonstrate a tendency to minimize response gaps with consistent timing across groups, fundamental principles of conversational turn-taking systems.

### Participation framework

We found that pauses, gaps, and phase responses had distinct temporal distributions, with gaps showing significantly shorter mean intervals than both pauses and phase responses. In humans, a comparable pattern is captured by the Simplest Systematics model^11,54^, which predicts that gaps between turns are shorter than pauses when self-selection occurs. Accordingly, short gaps in marmosets can also reflect the dyadic readiness to respond. Notably, phase responses, occurring when another individual’s vocalization is intercalated between two calls of the same marmoset (A-B-A), were longer than within-individual pauses, potential reflecting the additional processing time required to respond to another caller. These results are consistent with findings in other species, including harbor seal pups (*Phoca vitulina*)^55^, sperm whales (*Physeter macrocephalus*)^56^, zebra finches (*Taeniopygia guttata*)^51^, gibbons (*Hylobates lar*)^18^, and indris (*Indri indri*)^57^, which also show temporal adjustments or coordination during vocal interactions, suggesting that animals can modulate call timing based on social context. Our findings align with recent work in captive marmosets^27^, which emphasizes flexible timing, context-dependent turn transitions, and the combined influence of internal and external factors on turn-taking, rather than strict adherence to an internal rhythmic oscillator as proposed by Takahashi and colleagues^24^.

Although we did not directly measure cues such as gaze or body orientation, the temporal patterns observed may provide insight into the participation framework underlying animal, and specifically common marmoset, vocal interactions, as well as suggest new directions for future investigation of this turn-taking element. Previous studies show that common marmosets adjust call timing according to social context^35,58^, respond specifically to partners’ PHEEs^24,27^, and can direct vocalizations to specific individuals^59^. For instance, in humans, vocal cues such as timing, intonation, and speech rate can indicate turn transitions or signal the start of a new turn, even in the absence of visual cues^60^. Thus, our results may help improve our understanding of complex participation dynamics, highlighting parallels between common marmosets and turn-taking behaviors observed in humans and other non-human animals.

### Conclusions and limitations

Overall, wild common marmosets appear to exhibit a turn-taking system characterized by temporal regularities and structured coordination across various vocalizations and social contexts. These patterns persist even during intergroup encounters, where coordinated timing may help maintain group cohesion or reduce signal interference, highlighting the importance of examining beyond intragroup interactions. This should be taken into consideration in future studies, as current research is mostly focused on intragroup turn-taking^27,42,50^. Although some of the observed patterns could potentially arise from simple, non-cognitive processes, such as reactive timing or automatic responses, the combination of features we documented, including rapid turn transitions, relatively consistent gap intervals across groups, and non-random call-response sequences, parallels turn-taking patterns reported in other socially complex taxa, including birds^47^, non-primate mammals^15,46,61^, gibbons^18^, great apes^42,48,50^, and, to a lesser extent, humans^10^. However, the results of the present study should be interpreted with caution, as visibility and the behavioral context of interlocutors were not systematically included in the analyses, and these factors have been suggested to influence turn-taking dynamics in other communicative modalities, including gestural communication^27,48^. Nonetheless, this convergence suggests that structured temporal coordination may represent a shared feature of complex social communication, even if the underlying cognitive mechanisms vary across species^5,8,17^. Such regularities could emerge from a combination of attentional, perceptual, and social coordination mechanisms, providing insight into the potential evolutionary roots of conversational turn-taking. Comparative studies across diverse taxa using similar metrics will be important to further understand the functional significance and evolutionary trajectory of turn-taking systems.

## Methods

### Study site and groups

The study was conducted at the Baracuhy Biological Field Station (BBFS; S 7° 31′ 42″—W 36° 17′ 50″) in Cabaceiras, Paraíba, Northeast Brazil. The region is characterized by typical semiarid Caatinga vegetation, consisting of low, deciduous, and xerophytic (drought-adapted, thorny) shrubs interspersed with scattered small trees. The vegetation is generally sparse, with open areas and patches of denser shrubbery, reflecting the highly seasonal rainfall and prolonged dry periods typical of the region^62^. During the study period from July 2022 to March 2023, we followed four groups of common marmosets which consisted of a total of 25 individuals of different sexes and ages (see Table S6). We adopted age classes defined by Garber and colleagues^63^ for common marmosets: Infants (0–4 months), juveniles (5–11 months), subadults (12–15 months), and adults (≥ 15 months). Based on the assumption that full-fledged turn-taking skills of common marmosets are only present after the age of eight/nine months^26,35^, we observed and investigated vocal interactions of 18 subadult and adult animals only. All individuals were identified based on their body size, natural marks (e.g., size of the ear tufts or “star”), and/or scars^64^, and were fully habituated to the presence of human observers since 2013.

### Data collection

We observed individuals of all four groups for a total period of six months, eight days per month, and followed them throughout their entire activity period, from 5 a.m. to 5 p.m. We followed one group per day and used focal animal sampling method^65^, continuously recording one individual at a time for a 30-minute interval. We selected the focal subject randomly for each of the 30-minute intervals. That is, each animal was observed once, and after completing the 30-minute focal sampling recording for all subadults and adults in the group, we returned to the initial individual. We recorded the sampling frequency of each individual and, when choosing between multiple available individuals, prioritized those that had been sampled least often. We employed this method to ensure that individuals had similar observation hours. During these focal follows, we documented all vocal interactions between the focal animal and other individuals. Based on previous studies on vocalizations of primates^29,34^, we defined vocalizations as any sound produced by the vocal cords or vocal tract, involving laryngeal activity and respiratory movements. For the recording of vocal exchanges, we used an external unidirectional microphone (Sennheiser MKE 600), with a foam windshield, attached to a digital recorder (Tascam DR-100 mkII) at a 16-bit quantization and 48-kHz sampling rate^29^. The microphone was positioned 2 m away from the focal animal, and recordings were made throughout the 30-minute focal collection, with each recorded clip not exceeding five minutes in duration.

Whenever the focal animal produced a vocalization, we recorded via the microphone information regarding specific aspects of the observed interaction: (i) The identities of the interlocutors (e.g., the ID of the other individual(s) that replied vocally to the signal made by our focal and distinguished the initiator and the receiver, whenever possible) and (ii) the call type produced by both interlocutors.

### Ethics statement

In an effort to avoid influencing the natural behaviour of the individuals, our study was purely observational and non-invasive with audio recordings taken from a minimum distance of 2 m. The present study was conducted in accordance with the Brazilian laws for vertebrate animals, followed the recommendations of the ‘Animals (Scientific Procedures) Act 1986’, as published by the government of the United Kingdom, the principles of “Ethical Treatment of Non-Human Primates” as stated by the American Society of Primatologists, and was approved by the Ethics Committee for the Use of Animals of the Federal Rural University of Pernambuco (CEUA License No. 1712281122/2022) and SISBio (License No. 82271-1).

### Data extraction and coding

During a total of 540 h of focal observations of 18 individuals (median of direct observation: 25 h/individual, ± 4.1 h, range from 18 to 40 h), we recorded around 5820 audio files, which were transferred to a computer. We manually extracted all vocalizations and associated data, following a pre-established coding scheme, and using the program ELAN (version 6.7). From those, we selected 703 audio files (number of coded audio files used for the analysis per group: Hou: 125; Pom: 258; Coq: 141; Sws: 179) that were used based on: (i) audio quality (e.g., background noise, other animal vocalizations); (ii) the presence of vocalizations from marmosets; and (iii) vocalizations that could be clearly heard and identified (e.g., most agonistic interactions were excluded because the individuals producing the calls could not be reliably identified). We only coded vocalizations that met the following criteria: The call was (i) initiated by subadults or adults, or (ii) received a response from sub-adult or adult individuals. Following previous studies on vocalizations of common marmosets^29,34^, calls were divided into syllables (simple and single vocal elements, e.g., TRILL), sequence calls (sequences of one or more identical syllables made by the same individual, separated by less than 0.5 s from its following syllable, e.g., PHEE plus PHEE) and compound calls (sequences of one or more different syllables made by the same individual, separated by less than 0.5 s from its following syllable, e.g., TRILL plus PHEE^29,34^, please see supplementary material, for more information on the vocalization types used and their respective spectrograms, Table S1, Figure S1). To enable a comprehensive understanding of the diversity of vocalizations in our study individuals, we used a conservative approach and coded only vocal exchanges (turns) when one individual clearly produced a vocalization within ten seconds of another individual’s call (the onset of the preceding call needed to occur within 10 s of the offset of the first signal). This threshold was chosen based on prior work showing that over 90% of antiphonal calls in marmosets occur within 6–9 seconds^38,66^, and to align with similar response windows used in other studies of vocal communication in marmosets^24,27,29^. In addition, we visually compared the data using a 30-second, 10-second and 5-second threshold (see Supplementary Figure S7). The vast majority of responses occurred within the 10-second window when compared with the 30-second window, with only a few outliers extending beyond this interval. In contrast, using a 5-second window excluded a number of longer-latency responses, including interactions involving long calls such as PHEE calls. Based on these observations, we adopted the 10-second threshold for subsequent analyses, as it retained most biologically relevant responses while minimizing the inclusion of unrelated vocalizations.

#### Flexibility of turn-taking organization

To investigate this element, we examined whether common marmosets were able to voluntarily change or adjust a given vocalization^17,67^. For this, we extracted: (1) “Repetition,” repetition of a signal by the same individual, if there was no response with a minimum interval of 0.5 s and maximum interval of ten seconds between those signals; (2) “Repetition” was then divided into “Persistence” when the vocal signal was the same as the previous one (e.g., PHEE and PHEE) and “Elaboration” when animals changed the vocalization (e.g., PHEE and TWITTER^68^; and (3) “Turn-transition,” number of pair vocalizations (signal-response) made during the interaction with intervals no longer than ten seconds^17^.

#### Adjacency pair-like sequences

To investigate this element, we examined whether specific call types or sequences of vocalizations were responded to by the same call type or sequence by another individual^11,17^. To access this element, we coded: (1) the type of call made by the two interlocutors (in instances where a sequence call or a compound call was identified, we extended the coding to encompass the specific syllables within these vocalizations); and (2) the number of syllables within a sequence or compound call made by initiator and receiver^29,34^.

#### Temporal relationships

To investigate this element, we examined whether vocal exchanges of common marmosets were characterized by distinct temporal relationships. We operationalized this element via measuring (1) “Gaps” the time between two consecutive vocalizations made by different individuals from the offset of a first signal until the onset of its response^13,69^. This measure can include overlapping vocalizations, resulting in negative values when the response begins before the preceding call has ended.

#### Participation framework

Recent studies investigated this element by measuring gaze or body orientation^17,67^, however due to the small size of marmosets, their arboreal behavior and often not optimal visibility in the natural environment^28^, we operationalized this element by measuring “pauses,” “gaps” and “phase responses”^24^. For that we coded (1) “Pauses” the time between two consecutive vocalizations produced by the same individual, from the offset of the first call until the onset of the second call, separated by more than 0.5 seconds from the initiator’s next vocalization, and occurring within ten seconds (see further details, in the statistical analyses Sects^24,25^.; (2) “Gaps”; and (3) “Phase Responses” by measuring the time between two consecutive vocalizations (offset-onset) produced by the same individual when another individual calls in between^24,67,70^(e.g., Individual A calls, Individual B calls, then Individual A calls again; the Phase Response is the time from A’s first to second call). These measures allow us to assess how marmosets structure their participation in vocal interactions, capturing who is “taking a turn” and how timing adjusts when multiple individuals interact, even when visual cues are not available.

### Reliability

To ensure reliability of coded audio files, a second researcher blind to the research questions coded 12% of all audio-files (i.e., 80 out of 703) with a special focus on the parameters introduced above. We used the initiator and receiver ID and call type to calculate Cohen’s kappa. The analysis resulted in 0.97 and 0.89 of agreement for ID and vocalization type, respectively, which are “very good” levels of agreement^71^. Furthermore, we conducted an Intraclass Correlation Coefficient (ICC) analysis for gaps, as it is a continuous variable, and obtained a value of 0.84, which represents a “very good” level of agreement^72^.

### Statistical analyses

After coding all selected audio files, we transferred the data to Excel 2019 MSO (16.0.10827.20138). To investigate our three research objectives, we concentrated solely on the data where we were able to reliably identify at least one of the individuals within a dyadic interaction. We concentrated on the dyadic level for three main reasons: (1) previous studies on vocal interactions have employed a similar approach^73^; (2) observing interactions involving more than two wild individuals and obtaining reliable data is particularly challenging; and (3) prior research has shown that marmosets may alter their communicative behaviors depending on whether they are engaged in dyadic or triadic interactions^27^. Our first and second research aims were based solely on descriptive data, while the third research question was divided into the four main elements of turn-taking, as described below.

For the *flexibility of turn-taking organization element*, we analyzed it in two complementary ways. First, to investigate whether repetition type and frequency of repetitions affected the probability of eliciting a response, we fitted a Generalized Linear Mixed Model (GLMM) with a binomial family using *lme4*^74^ with repetition type (elaboration or persistence) and the number of repetitions as fixed effects. Initiator and interaction ID were included as nested random intercepts to account for the hierarchical structure. The response variable was whether a reply occurred or not. All analyses were restricted to the beginning of interactions, defined as events with no prior vocal exchange within the dyad for at least ten seconds. Second, using a bidirectional Markov Chain framework, we examined the sequential structure of vocal turn-taking interactions. Vocal sequences were classified into transient states representing the progression of signaling (S) and responding (R) vocalizations across interaction turns (e.g., S1: first signal produced by the initiator; R1: first response produced by the receiver; S2 and R2: second signaling and responding turns; S3 + and R3+: third or subsequent turns). Interactions terminated in the absorbing state Ceased, indicating the end of the vocal exchange. Transition probabilities between states were calculated to assess the likelihood of marmosets continuing repeating vocal signals, responding to, or terminating vocal interactions across successive turns. Additionally, we investigated whether common marmosets engaged in multiple turn-transitions during vocal interactions, a key feature of human conversational turn-taking^10^. To do so, we implemented a function to identify turn-transitions, defined as consecutive vocalizations from different individuals occurring within a maximum interval of 10 s. For the observed dataset, sequences of consecutive transitions were counted (ranging from one to six or more turns) and classified as either intra-group or inter-group. To evaluate whether these sequences occurred more often than expected by chance, we generated a null distribution based on 1,000 simulations in which calls were randomly reassigned to individuals and groups within each recording, and new start times were independently assigned, while preserving the total number of calls and total vocal duration. Our primary objective was to examine the capacity for coordinated vocal exchange between marmosets in wild settings. Each simulated dataset was processed using the same detection function, and observed frequencies were compared with the simulated distributions, separately for each group type and number of transitions, to obtain empirical p-values representing the proportion of simulated frequencies greater than or equal to the observed values. We additionally conducted all analyses on a reduced dataset including only individuals from identified groups and results were qualitatively identical and are reported in the supplementary (Figure S3, S4 and Table S2).

To investigate *adjacency pair-like sequences element*, we conducted two collocation analyses to assess whether specific call types produced by one individual (A) were more likely to be followed by specific responses from another individual (B)^75^. This involved examining call types for co-occurrences between initiator and receiver signals. The strength of each pair - indicating attraction or repulsion - was calculated by comparing observed co-occurrence frequencies with those expected by chance. We used Multiple Distinctive Collocation Analysis (MDCA), a method that statistically contrasts all possible bigram combinations using corrected binomial probability tests to determine whether certain call pairs occur significantly more or less frequently than expected^76^. Specifically, our objective was two-fold: (1) evaluate whether the same call type was paired with an exact same call type; (2) investigate if vocalization pairs also matched the number of syllables within a sequence. For the latter, we took one of the strongest collocational pairs found (PHEE-PHEE) that had been used in sequences by our study individuals and analyzed this data set using again MDCA. We investigated whether PHEE vocalizations with a specific number of syllables would prompt a response with PHEE vocalizations containing the same number of syllables.

To investigate the *temporal relationships element*, we first built a histogram with all the interval gaps between initiator and receiver and calculated the median. To assess whether temporal gaps of vocal exchanges remained consistent across observed vocalization pairs and between groups, we performed a GLMM with Gamma distribution and a log link function^74^. Because this distribution requires only positive values, we adjusted our data by adding a small constant to all observations and then applying a log transformation. This ensured that no zero values were included and that the data met the assumptions of the Gamma distribution. Fixed effects included vocalization pairs with strong collocational strength (e.g., PHEE–PHEE), group identity (unidentified groups and individuals not belonging to the known groups were excluded) and the duration of the first vocalization. The response variable was gap length. Initiator and interaction ID were included as nested random intercepts. Finally, we employed post-hoc comparisons using estimated marginal means (*emmeans*) to identify which pairs differed significantly. The comparisons were adjusted for multiple testing using the Tukey method to control the family-wise error rate. Since manual annotation precision varied slightly across coders, resulting in minor rounding values in some recordings when the waveform was not fully zoomed, we verified if these differences did not influence our analysis. Therefore, we conducted two complementary checks. First, we fitted a linear mixed model (LMM) on the full dataset using raw temporal gaps (without log transformation) and a simplified grouping variable (POM vs. Other groups). Second, we fitted a generalized linear mixed model (GLMM) on log-transformed gaps, using a Gamma distribution with a log link function, the same simplified grouping variable and group identity, and the duration of the first vocalization as covariates. We then compared this model to a null model without the grouping variable. All analyses confirmed that minor differences in annotation granularity across groups did not affect our main conclusions.

Finally, to investigate the *participation framework element*, we analyzed gaps, within-individual pauses, and phase responses of common marmosets. We fitted a Linear Mixed-Effects Model (LME) using the *lme4* package to test the effect of these categories on time (ms), with Category as fixed effect. Random intercepts were included for ID and interaction ID (nested structure) to account for repeated observations within individuals and interactions and Vocalization to account for potential dataset composition artifacts across different call types. All category values were retained as observed, without artificial truncation or transformation, in order to preserve their natural distribution. Moreover, post-hoc pairwise comparisons between categories were conducted using *emmeans* to determine which pairs differed significantly. The comparisons were adjusted for multiple testing using the Tukey method to control the family-wise error rate. This approach allowed us to examine whether within-individual pauses, between-individual gaps, and phase responses differed in their mean duration, in line with turn-taking studies in human and non-human primates, while accounting for the influence of the different call types^10,24,27,70^. Moreover, we also conducted a supplementary analysis including only fully identified individuals (excluding “Unk” cases) to assess whether phase responses could be affected by uncertain identities, and obtained qualitatively similar results (Table S7).

For all models, model selection was performed by comparing the full model to a null model (an intercept-only model with the same random and variance structures) using a likelihood ratio test. Model assumptions were validated by inspecting diagnostic plots of the residuals with the *DHARMa* package. In addition, we performed a leave-one-out sensitivity analysis to confirm that the model’s parameter estimates were not overly influenced by any single data point. We conducted all analyses using R (version 4.1.0 R Core Team 2021^77^) and considered results statistically significant if *p* ≤ 0.05.

## Supplementary Information

Below is the link to the electronic supplementary material.

Supplementary Material 1

## Data Availability

All data and analysis scripts underlying this study are deposited in Figshare. An anonymized link is provided here for peer review: https://figshare.com/s/3f189a8fb110de2840a5.
