# Supplementary material for: Hallmarks of social action in the vocal turn-taking of wild common marmosets (Callithrix jacchus)
Source: Sci Rep. 2026 Jul 2;16:20348. doi: 10.1038/s41598-026-60403-2 (PMC13328666; doi:10.1038/s41598-026-60403-2)
Supplement: Supplementary file 1 — Supplementary Material 1 [file 41598_2026_60403_MOESM1_ESM.docx]

**Supplementary Material**

**Hallmarks of social action in the vocal turn-taking of wild common marmosets (*Callithrix jacchus)***

**Table S1** Descriptions of the different vocalizations used for the analysis

| **Vocalization** | **Description** |
| --- | --- |
| PHEE | A single narrowband vocalization used either individually or in bouts ranging from one to six units, with intervals of up to 500 milliseconds between each unit (adapted from [1,2]). |
| TRILL | Low vocalization with sinusoidal FM structure uttered with the mouth almost closed [1,2]. |
| TWITTER | A wide-band vocalization with a series of short units [1,2]. |
| TRILL_PHEE* | Vocalization begins with a sinusoidal FM segment (Trill) that transitions into a gradually rising linear segment (Phee). It can be used either as a single units or in bouts, with all units adhering to a similar format [1]. |
| CHIRP* | Short, loud, and chirping vocalizations emitted in regularly spaced series of units [2,3]. |
| CHATTER* | Vocalization with Egg and Ock sounds given in intersperse series [2,4]. |
| PHEE_STRING* | Combination of two or more units of Phee vocalizations where the first vocalization of the sequence was always a Trill or a Trill_Phee vocalization (adapted from [1,2]). |
| SUBMISSIVE CRY* | Combination of submissive squeal along with Tsik and Trill vocalizations uttered in series [2]. |
| TWITTER_PHEE* | Combination of Twitter and Phee vocalizations without interval or maximum interval of 500 milliseconds between them [1]. |
| TRILL_TWITTER* | Combination of Trill and Twitter vocalizations without interval or maximum interval of 500 milliseconds between them [1]. |
| ALARM | Brief, sharp whistle made by the marmoset during a soft, “haa”-like exhalation [2]. |
| EGG | Short vocalization with a few harmonics [2]. |

*Vocalizations with the spectrogram for reference


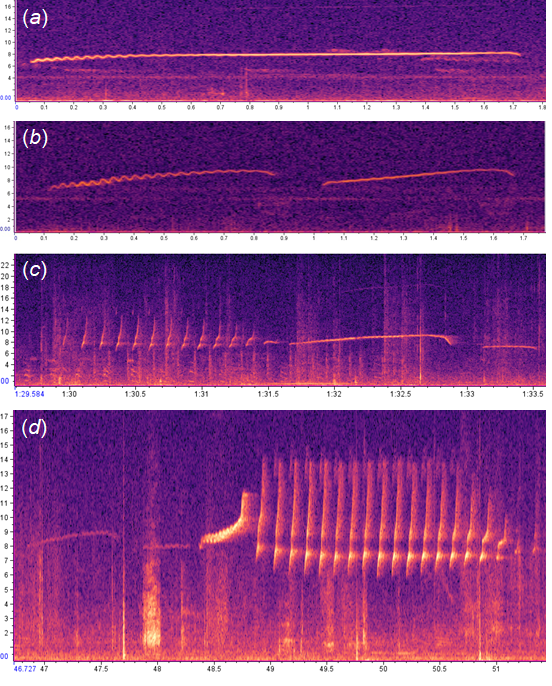

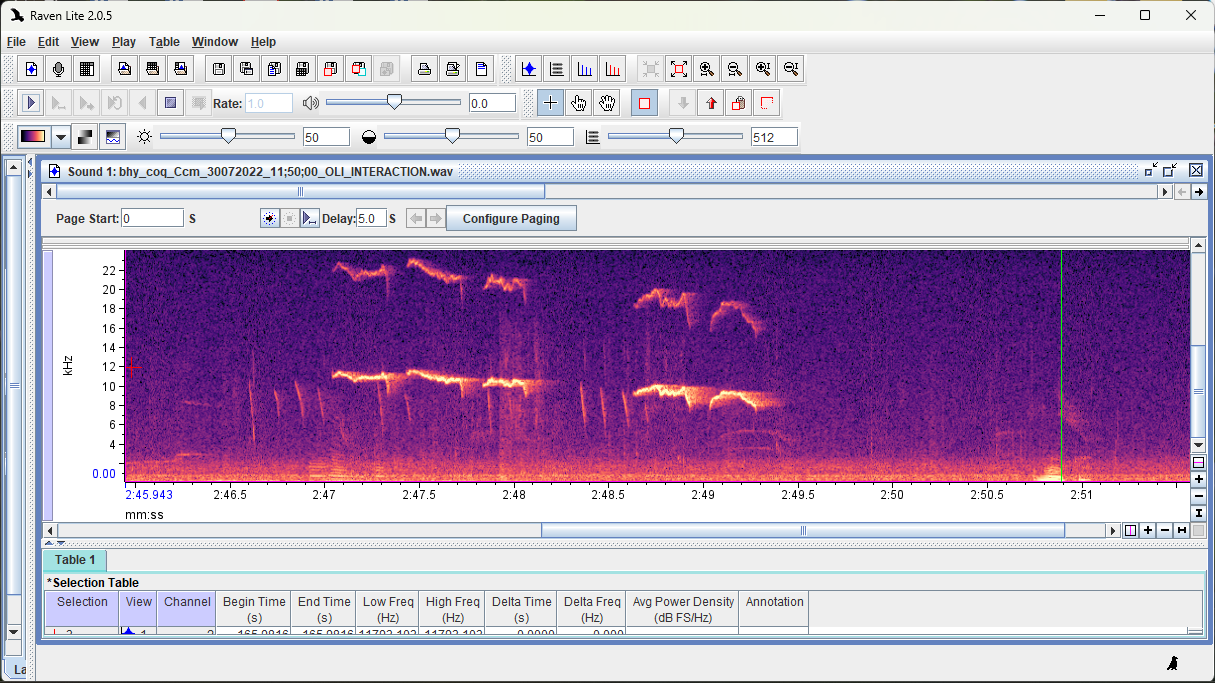


(e)


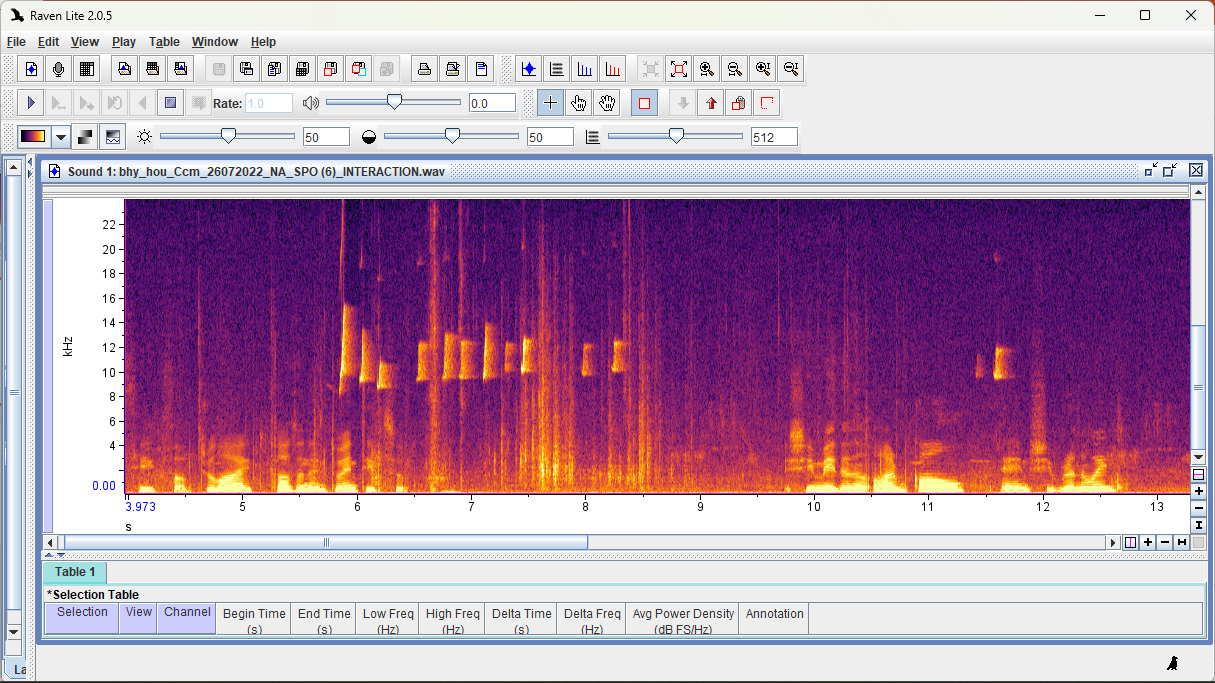


(f)


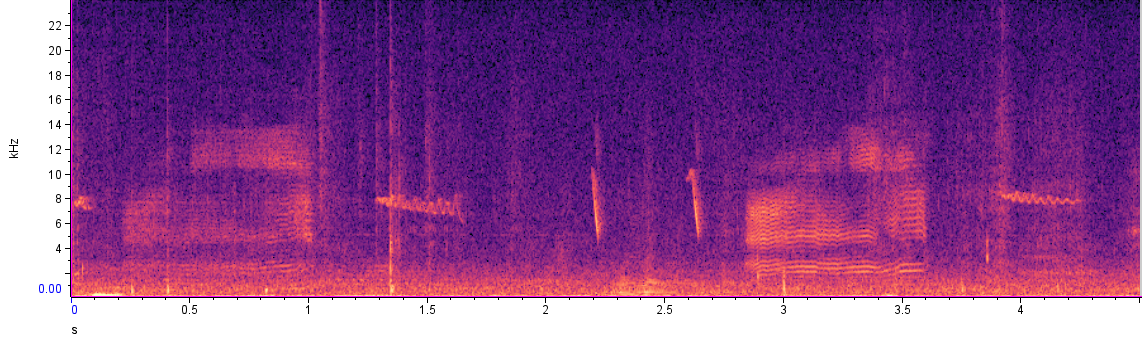


(h)


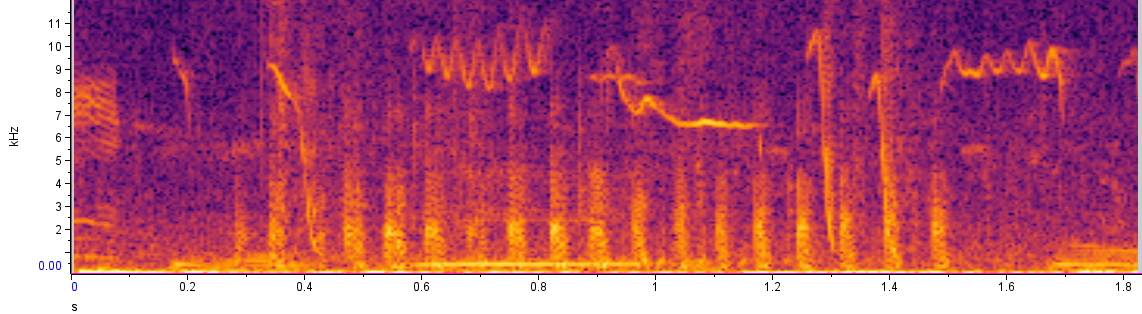


(i)


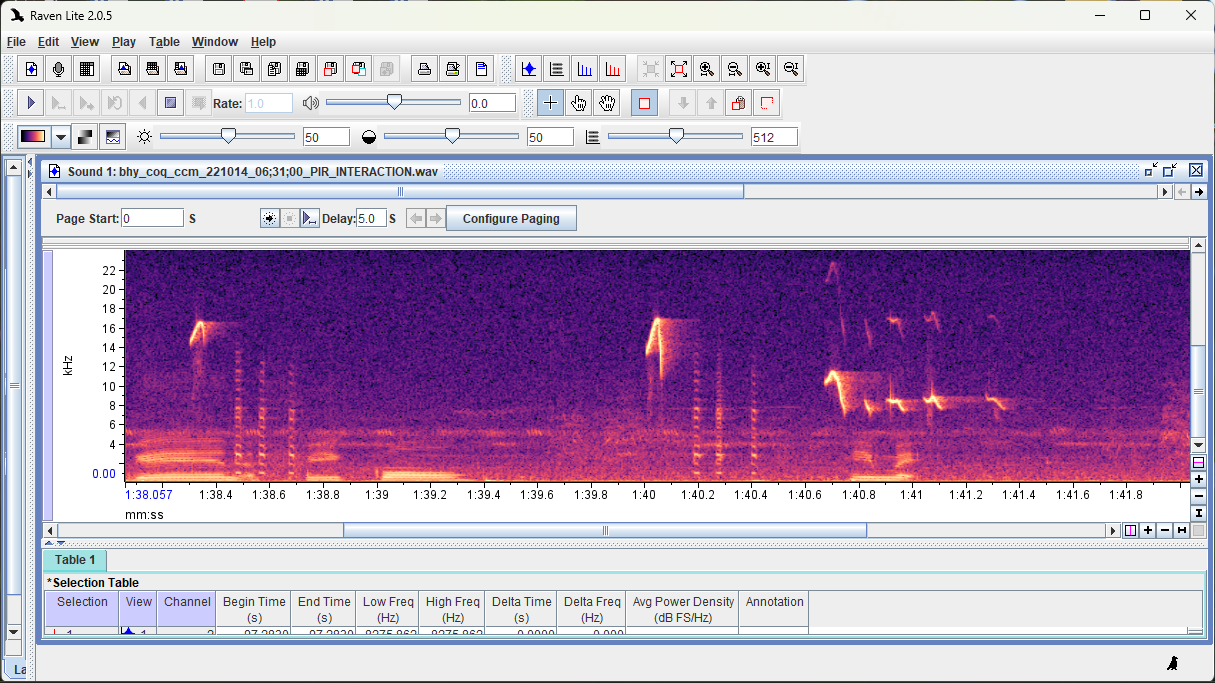


(g)

(f)

**Figure S1** Spectrograms of (a) Trill_Phee, (b) Phee_Strings, (c) Twitter_Phee, (d) Trill_Twitter, (e) Chirp, (f) Alarm, (g) Egg, (h) Submissive Cry and (i) Chatter vocalizations.


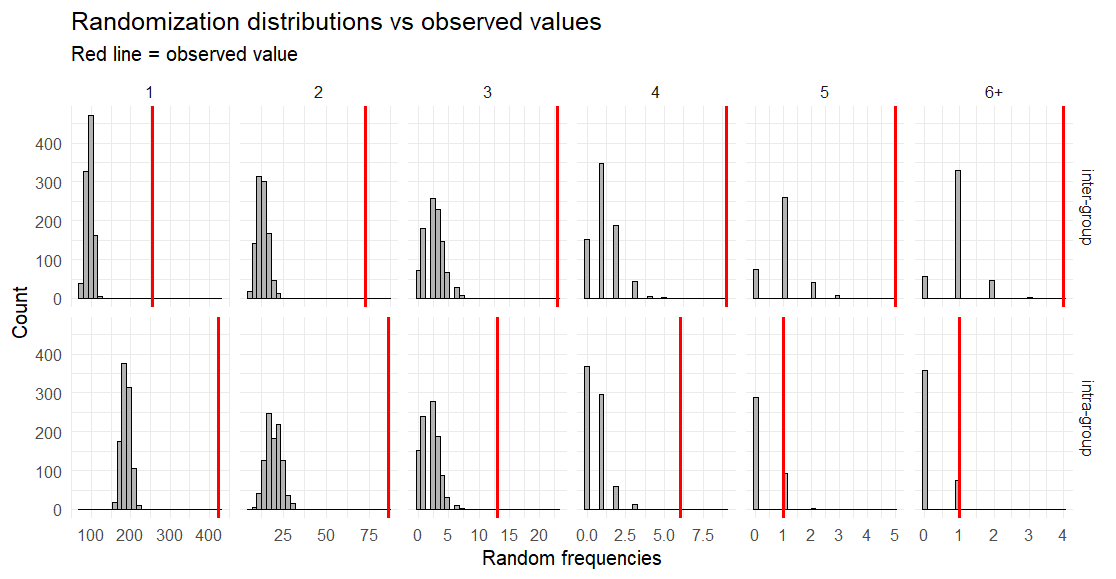


**Figure S2** Observed value vs. randomized distributions of turn-transitions in common marmoset during inter and intragroup vocal exchanges. Histograms represent the null distributions from 1,000 randomizations of turn-transitions and assignment across individuals and groups. Red vertical lines indicate observed frequencies of turn-transitions for intergroup (top panels) and intragroup (bottom panels) vocal interactions, separated by sequence length (1 to 6+ transitions). Observed values falling outside the null distribution indicate a higher-than-expected occurrence of multiple turn-transitions compared to chance.


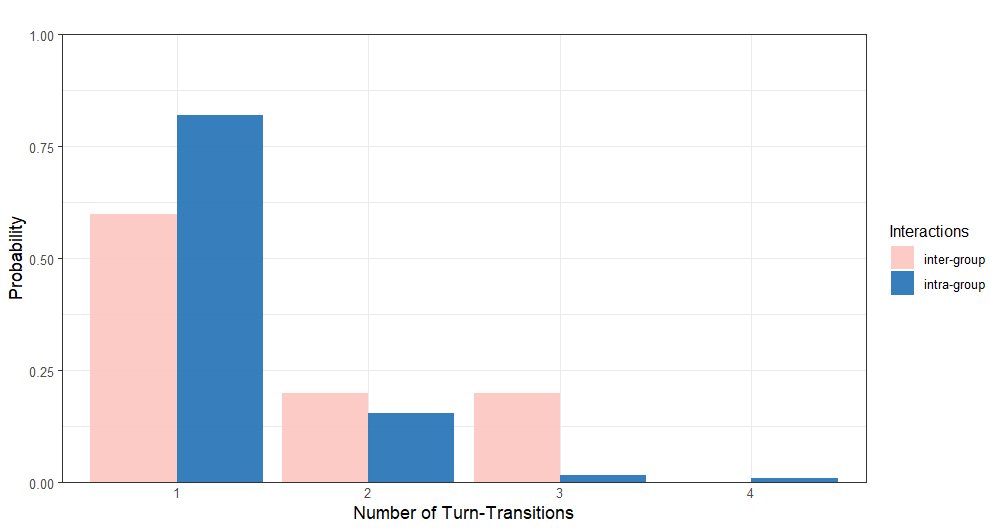


**Figure S3** Probability of turn-transitions ranging from a single transition (1) to more than six (6+) in vocal interactions, for both within-group (blue bars) and between-group (pink bars) interactions among individuals of fully identified groups.


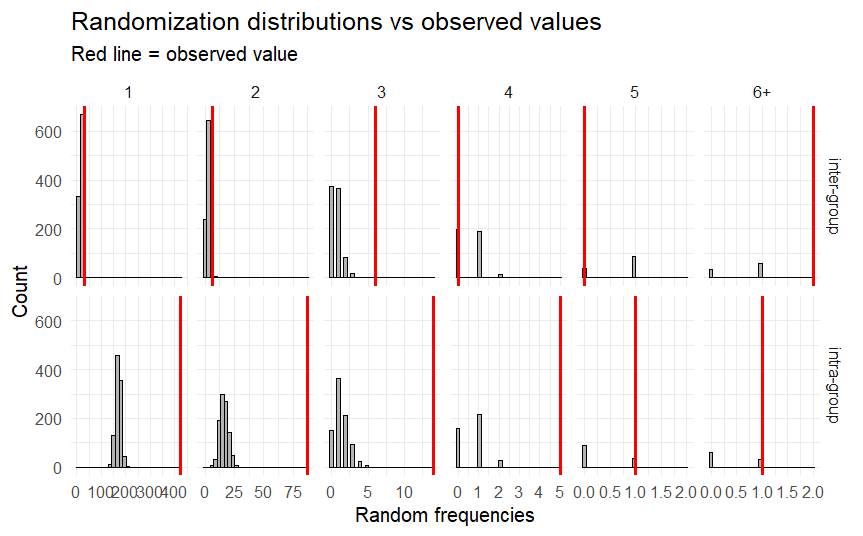


**Figure S4** Observed values versus randomized distributions of turn-transitions in common marmosets during inter- and intragroup vocal exchanges, including only interactions between individuals from fully identified groups. Histograms represent the null distributions from 1,000 randomizations of turn-transitions and assignment across individuals and groups. Red vertical lines indicate observed frequencies of turn-transitions for intergroup (top panels) and intragroup (bottom panels) vocal interactions, separated by sequence length (1 to 6+ transitions). Observed values falling outside the null distribution indicate a higher-than-expected occurrence of multiple turn-transitions compared to chance.

**Table S2** Comparison of results between the full dataset and the subset including only interactions between individuals from fully identified groups (First) and results of the simulations for both datasets (Second).

| Dataset | Group type | Turn-transitions | Frequency | Total | Probability |
| --- | --- | --- | --- | --- | --- |
| Full dataset | Inter-group | 1 | 255 | 369 | 0.691 |
| Full dataset | Inter-group | 2 | 73 | 369 | 0.198 |
| Full dataset | Inter-group | 3 | 23 | 369 | 0.062 |
| Full dataset | Inter-group | 4 | 9 | 369 | 0.024 |
| Full dataset | Inter-group | 5 | 5 | 369 | 0.014 |
| Full dataset | Inter-group | 6+ | 4 | 369 | 0.011 |
| Full dataset | Intra-group | 1 | 421 | 528 | 0.797 |
| Full dataset | Intra-group | 2 | 86 | 528 | 0.163 |
| Full dataset | Intra-group | 3 | 13 | 528 | 0.025 |
| Full dataset | Intra-group | 4 | 6 | 528 | 0.011 |
| Full dataset | Intra-group | 5 | 1 | 528 | 0.002 |
| Full dataset | Intra-group | 6+ | 1 | 528 | 0.002 |
| Identified-only dataset | Inter-group | 1 | 30 | 44 | 0.682 |
| Identified-only dataset | Inter-group | 2 | 6 | 44 | 0.136 |
| Identified-only dataset | Inter-group | 3 | 6 | 44 | 0.136 |
| Identified-only dataset | Inter-group | 4 | 0 | 44 | 0.000 |
| Identified-only dataset | Inter-group | 5 | 0 | 44 | 0.000 |
| Identified-only dataset | Inter-group | 6+ | 2 | 44 | 0.046 |
| Identified-only dataset | Intra-group | 1 | 424 | 532 | 0.797 |
| Identified-only dataset | Intra-group | 2 | 87 | 532 | 0.164 |
| Identified-only dataset | Intra-group | 3 | 14 | 532 | 0.026 |
| Identified-only dataset | Intra-group | 4 | 5 | 532 | 0.009 |
| Identified-only dataset | Intra-group | 5 | 1 | 532 | 0.002 |
| Identified-only dataset | Intra-group | 6+ | 1 | 532 | 0.002 |

| Dataset | Group type | Turn-transitions | Simulations | p-value |
| --- | --- | --- | --- | --- |
| Full dataset | Inter-group | 1 | 1000 | **0.001** |
| Full dataset | Inter-group | 2 | 1000 | **0.001** |
| Full dataset | Inter-group | 3 | 992 | **0.001** |
| Full dataset | Inter-group | 4 | 777 | **0.001** |
| Full dataset | Inter-group | 5 | 401 | **0.002** |
| Full dataset | Inter-group | 6+ | 454 | **0.002** |
| Full dataset | Intra-group | 1 | 1000 | **0.001** |
| Full dataset | Intra-group | 2 | 1000 | **0.001** |
| Full dataset | Intra-group | 3 | 992 | **0.001** |
| Full dataset | Intra-group | 4 | 777 | **0.001** |
| Full dataset | Intra-group | 5 | 401 | 0.246 |
| Full dataset | Intra-group | 6+ | 454 | 0.207 |
| Identified-only dataset | Inter-group | 1 | 1000 | **0.002** |
| Identified-only dataset | Inter-group | 2 | 1000 | **0.048** |
| Identified-only dataset | Inter-group | 3 | 846 | **0.001** |
| Identified-only dataset | Inter-group | 4 | 404 | 1.000 |
| Identified-only dataset | Inter-group | 5 | 129 | 1.000 |
| Identified-only dataset | Inter-group | 6+ | 93 | **0.011** |
| Identified-only dataset | Intra-group | 1 | 1000 | **0.001** |
| Identified-only dataset | Intra-group | 2 | 1000 | **0.001** |
| Identified-only dataset | Intra-group | 3 | 846 | **0.001** |
| Identified-only dataset | Intra-group | 4 | 404 | **0.002** |
| Identified-only dataset | Intra-group | 5 | 129 | 0.300 |
| Identified-only dataset | Intra-group | 6+ | 93 | 0.372 |


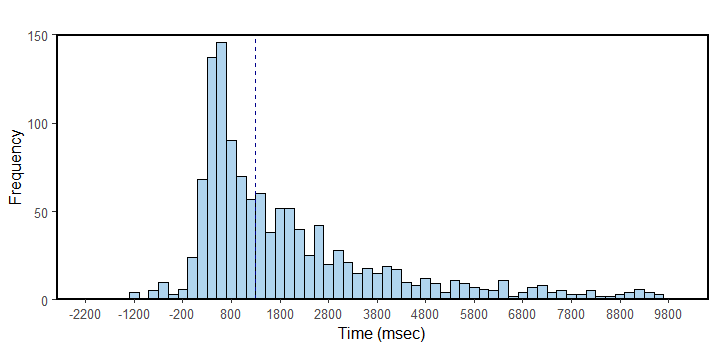


**Figure S6** Frequency distribution of gaps during vocal interactions. The dash blue line represents the median value of gaps.

**Table S4** GLMM results for predictors of gaps in common marmosets

| Predictor/Random Effect | Estimate | Standard Error | t value | p value | 2.5 % CI | 97.5 % CI |
| --- | --- | --- | --- | --- | --- | --- |
| *Fixed Effects* |  |  |  |  |  |  |
| (Intercept) | 2.088 | 0.047 | 88.07 | < 0.001 | 2.041 | 2.134 |
| Hou_Hou | 0.019 | 0.050 | 0.79 | 0.703 | -0.028 | 0.066 |
| Pom_Pom | -0.010 | 0.046 | -0.47 | 0.822 | -0.053 | 0.033 |
| Sws_Sws | -0.009 | 0.049 | -0.33 | 0.857 | -0.056 | 0.038 |
| Trill_Trill | -0.103 | 0.030 | -6.58 | **< 0.001** | -0.134 | -0.073 |
| Twitter_Twitter | -0.072 | 0.016 | -4.33 | **< 0.001** | -0.105 | -0.040 |
| Duration | 1.005e-06 | 4.251e-06 | 0.46 | 0.813 | -3.3e-06 | 5.3e-06 |
| *Random Effects* | Variance | Std. Dev. |  |  |  |  |
| ID_interaction:ID | 0.000 | 0.000 |  |  |  |  |
| ID | 0.000 | 0.000 |  |  |  |  |

**Table S5** LME results for predictors of vocal timing categories (gaps, pauses, phase responses) controlling for vocalization type, in common marmosets

| Predictor/  Random Effect | Estimate | Standard Error | t value | 2.5 % CI | 97.5 % CI |
| --- | --- | --- | --- | --- | --- |
| *Fixed Effects* |  |  |  |  |  |
| (Intercept) | 2048.9 | 315.0 | 6.505 | 1431.497 | 2666.229 |
| Pauses | 2937.5 | 185.6 | 15.828 | 2573.758 | 3301.275 |
| Phase_Response | 5229.5 | 161.4 | 32.391 | 4913.052 | 5545.910 |
| *Random Effects* | Variance | Std. Dev. |  |  |  |
| ID_interaction:ID | 1404302 | 1185.0 |  |  |  |
| ID | 26210 | 161.9 |  |  |  |
| Vocalization | 637154 | 798.2 |  |  |  |
| Residual | 6052179 | 2460.1 |  |  |  |

**Table S6** Overview of study individuals as a function of name, group affiliation, sex, age class and age (birth date in month and years).

| Name | Group | Sex | Age Class (Birth date) |
| --- | --- | --- | --- |
| Verde* | Hou¥ | Male | Adult |
| Estranha* | Hou¥ | Female | Adult |
| Spotty* | Hou¥ | Female | Adult |
| Rey* | Hou¥ | Male | Adult |
| Hansel** | Hou¥ | Male | Juvenile (December 2021) |
| Gretel** | Hou¥ | Female | Juvenile (December 2021) |
| Baby | Hou¥ | Male | Infant (May 2022) |
| Nala* | Pom¥ | Female | Adult |
| Scar* | Pom¥ | Male | Adult |
| Simba* | Pom¥ | Male | Adult |
| Pumba | Pom¥ | Female | Juvenile (March 2022) |
| Timon | Pom¥ | Female | Juvenile (March 2022) |
| Pirate* | Coq‡ | Male | Adult |
| Olivia* | Coq‡ | Female | Adult |
| Grey* | Coq‡ | Female | Adult |
| Black* | Coq‡ | Male | Adult |
| Lin** | Coq‡ | Female | Juvenile (November 2021) |
| Bluey | Coq‡ | Male | Infant (May 2022) |
| Bingo | Coq‡ | Female | Infant (May 2022) |
| Leia* | Sws‡ | Female | Adult |
| Monica* | Sws‡ | Female | Adult |
| Anakin* | Sws‡ | Male | Adult |
| Luke* | Sws‡ | Male | Adult |
| Yoda | Sws‡ | Female | Infant (April 2022) |
| Obi | Sws‡ | Female | Infant (April 2022) |

* refers to focal animals

** refers to individuals that were focal animals as soon as they entered the age of sub-adults.

Ages at the beginning of the study period (July 2022)

¥ neighboring groups (Hou and Pom)

‡ neighboring groups (Coq and Sws)

**
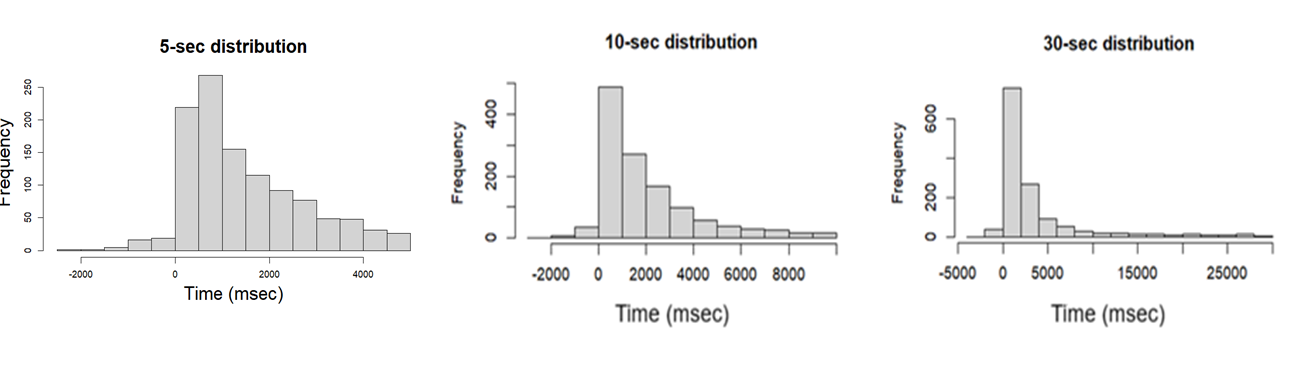
**

**Figure S7** Distributions of temporal values using, 5-second (left), 10-second (middle) and 30-second (right) thresholds in common marmoset vocal interactions.

**Table S7** LME results for predictors of vocal timing categories (gaps, pauses, phase responses) controlling for vocalization type, using only fully identified individuals (excluding “Unk” cases) in common marmosets.

| Predictor / Random Effect | Estimate | Standard Error | t value | 2.5 % CI | 97.5 % CI |
| --- | --- | --- | --- | --- | --- |
| *Fixed Effects* |  |  |  |  |  |
| (Intercept) | 2061.4 | 326.1 | 6.321 | 1422.233 | 2700.50 |
| Pauses | 2958.4 | 184.3 | 19.80 | 2597.206 | 3319.583 |
| Phase_Response | 5416.5 | 191.1 | 28.343 | 5041.954 | 5791.074 |
| *Random Effects* | Variance | Std. Dev. |  |  |  |
| ID_interaction:ID | 1122843 | 1059.6 |  |  |  |
| ID | 31019 | 176.1 |  |  |  |
| Vocalization | 704653 | 839.4 |  |  |  |
| Residual | 6124982 | 2474.9 |  |  |  |

**References**

1. Agamaite JA, Chang C-J, Osmanski MS, Wang X. 2015 A quantitative acoustic analysis of the vocal repertoire of the common marmoset (*Callithrix jacchus*) . *J. Acoust. Soc. Am.* **138**, 2906–2928. (doi:10.1121/1.4934268)

2. Bezerra BM, Souto A. 2008 Structure and usage of the vocal repertoire of *Callithrix jacchus*. *Int. J. Primatol.* **29**, 671–701. (doi:10.1007/s10764-008-9250-0)

3. Vitale A, Zanzoni M, Queyras A, Chiarotti F. 2003 Degree of social contact affects the emission of food calls in the common marmoset (*Callithrix jacchus*). *Am. J. Primatol.* **59**, 21–28. (doi:10.1002/ajp.10060)

4. Poole TB, Stevenson MF. 1976 An ethogram of the common marmoset (*Calithrix jacchu*s *jacchus*): general behavioural repertoire. *Anim. Behav.* **24**, 428–51.
